# Employment conditions and mental health of overseas female migrant domestic workers in Hong Kong: a parallel mediation analysis

**DOI:** 10.1186/s12939-024-02098-3

**Published:** 2024-01-17

**Authors:** Timothy S. Sumerlin, Jean H. Kim, Alvin Yik-Kiu Hui, Dicken Chan, Tim Liao, Sabu Padmadas, Eric Fong, Roger Y. Chung

**Affiliations:** 1 The Jockey Club School of Public Health and Primary Care, The Chinese University of Hong Kong, Hong Kong SAR, China; 2 School for Policy Studies, University of Bristol, Bristol, UK; 3 Department of Sociology, State University of New York Stony Brook, Brookhaven, NY USA; 4 Social Statistics and Demography, University of Southampton, Southampton, UK; 5 Department of Sociology, The University of Hong Kong, Hong Kong SAR, China; 6 Institute of Health Equity, The Chinese University of Hong Kong, Hong Kong SAR, China; 7 Centre for Bioethics, The Chinese University of Hong Kong, Hong Kong SAR, China

**Keywords:** Migrant domestic workers, Ethnic minority, Mental health, Employment conditions, Occupational health, Hong Kong

## Abstract

**Background:**

Female migrant domestic workers (MDW), approximately 8.5 million globally, often live in their employer’s home under vulnerable conditions. In Hong Kong, MDWs currently comprise 5% of the population. This study was conducted to assess the association between employment conditions and mental health, and the mediating roles stress and job satisfaction have, among female MDWs in Hong Kong.

**Methods:**

Participants completed an online cross-sectional survey. A total of 1,965 survey were collected between August 2020 and August 2021. Questions in the survey were related to MDWs background information, employment conditions, stress, job satisfaction, and two mental health outcomes: anxiety and depression. An employment conditions score was created to assess the cumulative effect poor employment conditions had on mental health. A multicategorical parallel mediation analysis was used to assess the direct effect employment conditions have on mental health and the indirect effects through stress and job satisfaction.

**Results:**

Overall, 17.7% of MDWs were reported to be suffering from anxiety and 30.8% from depression. An increase in poor employment conditions was statistically associated with an increase in both outcomes, while stress levels and job satisfaction mediated this association.

**Conclusions:**

The findings call for increased scrutiny of employment conditions and mental well-being of MDWs.

**Supplementary Information:**

The online version contains supplementary material available at 10.1186/s12939-024-02098-3.

## Background

International migrant labor continues to grow globally from 164 million in 2017 to 169 million in 2019 [1]. These migrant workers generally experience dislocation and isolation from their families and work in harsh conditions to earn income and send remittances to their left-behind families. They also commonly face a range of individual social and economic vulnerabilities due to their alien status in host countries, including precarious working and living conditions, and being at risk of poor mental health outcomes [2, 3]. International migrant workers comprise a heterogeneous mix, and they are often exposed to different and multiple risk factors affecting mental health. Globally, there are approximately 8.5 million female migrant domestic workers (MDW), and unlike other migrant workers, they are often subject to a “live-in” requirement where they are left with no choices but live and work in the home of their employers [4, 5]. The home as both workplace and living space provides little time away from the employer and their family, thus increasing personal vulnerabilities such as abuse and subjugation [6]. It is therefore imperative to understand how their unique employment circumstances may contribute to their mental health and well-being outcomes.

Among mental health disorders, anxiety is characterized as a future oriented mood where one expects negative outcomes, and can be accompanied with symptoms including worry, avoidance, and muscle tension [7, 8]. Depression is another common condition defined as “persistent sadness and a lack of interest or pleasure in previously rewarding or enjoyable activities” [9]. While the two conditions differ from each other, they usually share symptoms of negative affect and a feeling of distress [10]. In 2019, the estimated global incidence of anxiety disorders was 301 million, while depression affected an estimated 280 million making it the single largest contributor to years of life lost to disability [11, 12]. Given the unique live-in nature of many MDWs and thus the unconventional relationship between employee and employer, various employment conditions are an area of interest when assessing the mental health of MDWs.

MDWs are known to experience poor employment conditions, often characterized into four main areas: (1) their physical environment (inadequate sleeping conditions and lack of privacy), (2) overwork and exploitation (long working hours and being asked to do dangerous/illegal work), (3) verbal, physical, and sexual abuse, and (4) material deprivation (inadequate wages and lack of food) [6, 13–15]. These conditions have been independently associated with increased risk of poor mental health outcomes [6, 13–15]. However, a cumulative effect of experiencing poor employment conditions has not been systematically investigated. Furthermore, previous studies often relied on small sample size [6, 13, 15], and focused on mental health in a general sense rather than specific conditions such as anxiety or depression [6, 14, 15]. On the other hand, existing literature have often overlooked the wider physical environment in which MDWs live and work. Finally, the mechanisms through which employment conditions can affect mental health have not been systematically examined among MDWs. Identifying the mechanisms that lead to poor mental health in MDWs can help public health researchers and community health workers create targeted interventions. This study thereby aims to use mediation analysis to evaluate pathways between employment conditions and mental health that may be readily addressable.

Among workers in general, increases in anxiety and depression have been associated with a decrease in work productivity [16]. Within the context of employment, both stress and job dissatisfaction have both been linked to increased poor mental health [17–21]. Additionally, the work environment has been repeatedly associated with mood disorders, which calls for the need to improve working environments to enhance mental health [22]. Furthermore, both stress and job satisfaction are well-studied factors which can be addressed by interventions to improve mental health [23–25]. We hypothesize that an increase in poor employment conditions among MDWs is associated with an increase in their stress and decrease job satisfaction, and in turn increase both anxiety and depressive symptoms.

In 2020, the Hong Kong SAR region of China hosted 368,217 female MDWs in 2020, representing 5% of the 7.4 million city’s residents [26]. Females accounted for 98.5% of all MDWs, with 55% from the Philippines and 43% from Indonesia [26]. As per the government regulations, all MDWs must enter Hong Kong under the same visa scheme which allows them to only work as a MDW for one family whom they also must live with (i.e., the “live-in” requirement) [4]. Unlike other migrants in Hong Kong, MDWs cannot seek permanent residency [27]. Yet, MDWs are an inevitable and integral part of the local economies. In Hong Kong, MDWs accounted for 9.3% of the overall workforce in 2016, and their presence enabled local women to increase their labor force participation [28]. In addition to domestic household chores, they are dispensed to function as caretakers of children, older adults, and people with disability.

**Fig. 1:**
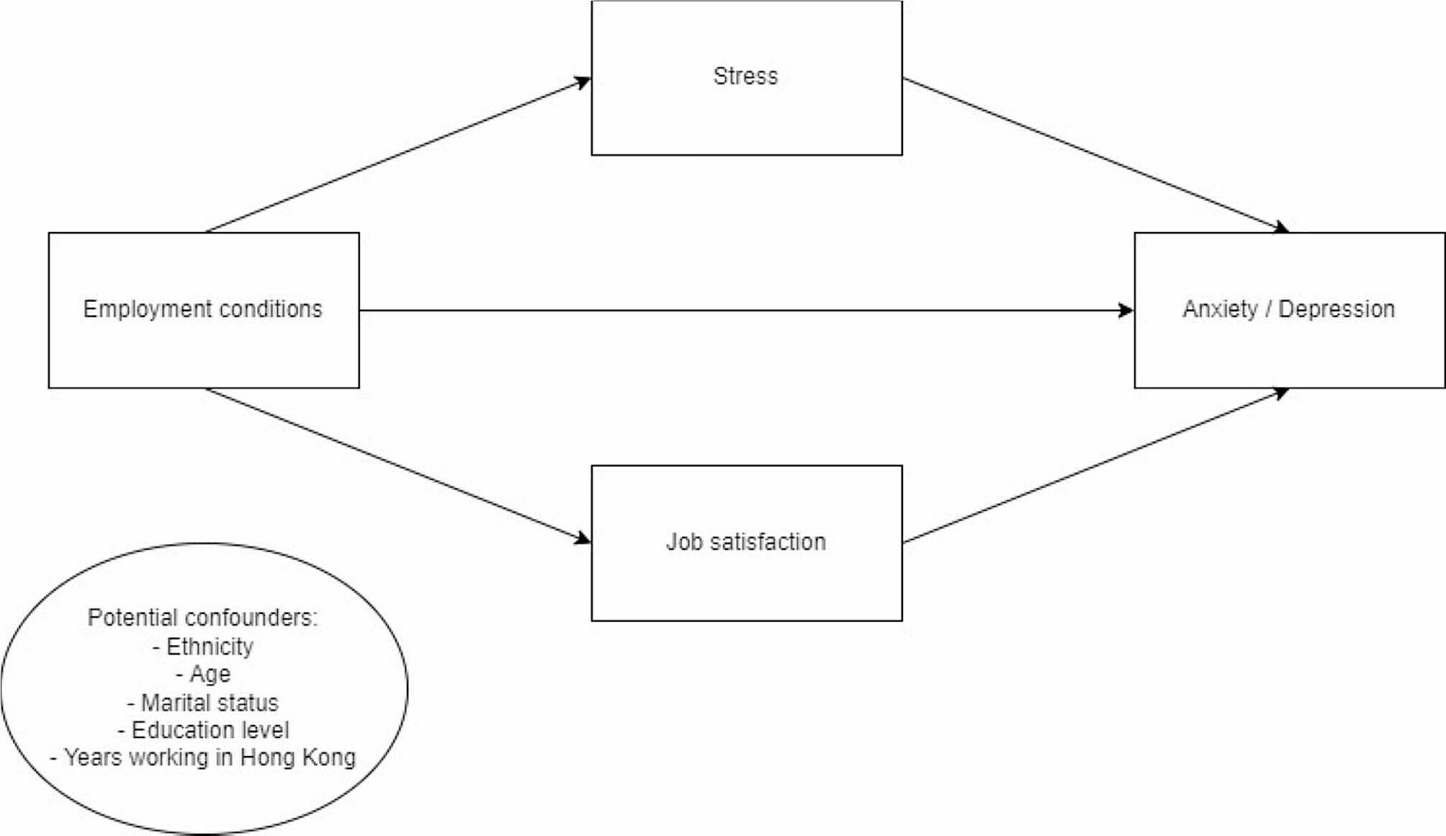
Conceptual Diagram

The present research adapts the WHO Employment Conditions and Health Inequality Framework which considers the impact of employment conditions on health inequalities [29]. As the Hong Kong MDWs in this study are all exposed to the same macro-level factors (i.e., labor policy), this study will focus on the micro-level employment conditions that can vary widely from each MDW. This framework views individual background and employment conditions as independent factors contributing to mental health outcomes, mediated by psychosocial factors (e.g., stress, job satisfaction). Based on the framework (Fig. 1), this study seeks to examine the association between employment conditions with anxiety and depressive symptoms as outcomes among female MDWs in Hong Kong. We will also examine the possible mediating role of stress and job satisfaction in this relationship.

## Methods

### Study setting and data collection

Due to COVID restrictions, we relied on an online cross-sectional survey for this research. The sample inclusion criteria included: (1) female MDWs from the Philippines or Indonesia, and (2) minimum one-year work history in Hong Kong. The survey was offered in both English and Indonesian (Bahasa) languages. The original English version was translated into Indonesian, back-translated, and then examined for equivalence. Pilot testing with cognitive debriefing was conducted on 50 Filipino and Indonesian MDWs to ensure comprehensibility of the instrument and where appropriate, a few questions were removed or refined for clarity. Data were collected from August 2020 to August 2021 using a novel multi-stage cluster random sampling approach designed specifically for this study population [30]. MDWs were initially approached in public areas on Sunday, their day off, when many MDWs gather in groups. Those who met the inclusion criteria were allowed to complete an online survey which was used in order to minimize face-to-face contact during the COVID-19 pandemic.

However, social distancing restrictions implemented during the COVID-19 pandemic prohibited public gatherings. To reach study respondents, a probability sampling method was supplemented with a non-probability sampling. MDW influencers in Hong Kong assisted in distributing the survey link to a large Facebook group and WhatsApp group of the local MDW community. Each participant first answered questions related to the inclusion criteria before being allowed to continue to complete the survey. All participants received a 100 HKD (about 12.75 USD) cash coupon as an incentive upon completion of the survey. On average, the survey took forty minutes to complete. Written informed consent was obtained prior to survey administration and ethics approval was obtained by the Survey and Behavioural Research Ethics Committee of The Chinese University of Hong Kong [Ref No. 24610417]. The STROBE checklist was completed to ensure comprehensive reporting of the study.

### Sample size calculation

Sample size was calculated considering a 3% margin of error, a 95% confidence interval, a 50% response distribution, and a population size of roughly 370,000, requiring a sample size of at least 1,065 respondents.

### Employment conditions score

Questions on employment conditions were asked addressing 17 items on MDW’s physical environment at home/work, workload, material circumstances such as adequate food and wages, and their experiences on exploitation and abuse by employers (See Table 1). A summative employment conditions score (ECS) was computed (scored 0 to 17) whereby a higher score indicated poorer employment conditions (Cronbach’s alpha = 0.73 for the 17-item scale). The Spearman’s correlation coefficients between each of the items can be found in Supplementary Table 1. Based on the distribution of the ECS, respondents were grouped into three levels of working conditions. Those whose ECS was less than the interquartile range (IQR) (those reporting no adverse work conditions) were classified as having “good” working conditions. Those in the interquartile range were classified as having “average” working conditions while those reporting ECS greater than IQR were classified as having “poor” working conditions.

**Table 1:** Characteristics of the employment conditions score (*n* = 1965)

	Frequency		Anxiety	Depression
	n	%	Mean (SD)	Mean (SD)
**Employment conditions score (0–17) median (IQR)**	2 (1, 3)			
Good (0)	490	24.9%	1.6 (2.5)	2.2 (2.8)
Average (1–3)	940	47.8%	2.9 (3.5)	3.8 (3.8)
Poor (≥ 4)	535	27.2%	4.8 (4.5)	5.8 (4.5)
**Items included in the employment conditions score**:				
1) Sleeping arrangement				
Private room/share room	1871	95.2%	3.0 (3.7)	3.9 (3.9)
Common living space	94	4.8%	4.3 (4.5)	5.4 (4.9)
2) At your employer’s home, is there overcrowding or lack of privacy?				
No	1436	73.1%	2.7 (3.5)	3.5 (3.8)
Yes	530	26.9%	4.2 (4.3)	5.2 (4.2)
3) At your employer’s home, is it unsanitary, pest infested, or poorly ventilated?				
No	1565	79.6%	2.8 (3.6)	3.7 (3.8)
Yes	400	20.4%	4.2 (4.3)	5.1 (5.0)
4) At your employer’s home, is there any lack of water, electricity, or plumbing?				
No	1737	88.4%	3.0 (3.7)	3.8 (3.9)
Yes	229	11.6%	4.1 (4.4)	5.1 (4.6)
5) Do you receive a food allowance or have food provided?				
Yes	1923	97.9%	3.1 (3.7)	3.9 (3.9)
No	42	2.1%	4.8 (5.2)	6.1 (5.4)
6) Did you always get enough food to eat?				
Yes	1492	75.9%	2.7 (3.6)	3.6 (3.8)
No	473	24.1%	4.2 (4.1)	5.1 (4.3)
7) Did you ever have your salary withheld or given less?				
No	1689	86.0%	2.9 (3.7)	3.8 (3.9)
Yes	276	14.0%	4.0 (4.2)	5.0 (4.5)
8) Did you ever have your phone or internet taken away?				
No	1778	90.5%	3.0 (3.7)	3.8 (3.9)
Yes	187	9.5%	4.1 (4.0)	5.3 (4.2)
9) Did your employer ever verbally abuse you?				
No	1653	84.1%	2.7 (3.5)	3.5 (3.8)
Yes	312	15.9%	5.1 (4.6)	6.2 (4.3)
10) Did your employer ever physically or sexually abuse you?				
No	1904	96.9%	3.0 (3.7)	3.9 (3.9)
Yes	61	3.1%	5.1 (5.5)	6.0 (5.0)
11) Did your employer ever take away your day off?				
No	1664	84.7%	2.9 (3.6)	3.6 (3.8)
Yes	301	15.3%	4.3 (4.4)	5.6 (4.5)
12) Did your employer give you all statutory holidays?				
Yes	1869	95.1%	3.0 (3.7)	3.8 (3.9)
No	96	4.9%	4.4 (4.9)	6.1 (4.8)
13) Did your employer ever restrict you from going outside?				
No	1749	89.0%	3.0 (3.7)	3.8 (3.9)
Yes	216	11.0%	4.2 (4.4)	5.5 (4.5)
14) Did your make you do work beyond what is stated in the contract?				
No	1834	93.3%	3.0 (3.7)	3.8 (3.9)
Yes	131	6.7%	4.9 (4.5)	6.1 (4.7)
15) Past month average daily working hours				
≤ 15 h	1556	79.2%	2.8 (3.5)	3.6 (3.7)
≥ 16 h	409	20.8%	4.3 (4.4)	5.2 (4.6)
16) Were you ever woken in the middle of the night and asked to work?				
No	1509	76.8%	2.7 (3.5)	3.5 (3.7)
Yes	456	23.2%	4.4 (4.4)	5.5 (4.4)
17) Did you ever have to work before being allowed to begin your day off?				
No	1338	68.1%	2.7 (3.5)	3.4 (3.8)
Yes	628	31.9%	4.0 (4.1)	5.0 (4.2)

Employment conditions score Cronbach’s alpha = 0.73

### Mediator measures

Job satisfaction was measured using the 10-item Generic Job Satisfaction Scale (GJSS), which asks about work aspects including job security, recognition, compensation and interpersonal relationships using a 5-point Likert scale (strongly disagree to strongly agree) [31]. The summative score ranges from 10 to 50 with a higher score indicating better job satisfaction. GJSS has been previously shown as a reliable measure of job satisfaction in various occupations and used to assess job satisfaction of female MDWs [24, 31]. The standardized Cronbach’s alphas for GJSS in this survey were 0.92 and 0.94 in the English and Indonesian versions, respectively.

Stress was asked as a single-item question of “do you feel stressed these days?” on a 5-point Likert scale from “not at all” to “very much” [32]. The item was assessed as a continuous variable (1–5) with a higher value indicating more stress. This question has been previously shown to be a valid and reliable measure of stress [32, 33].

### Background characteristics

Questions related to the respondents’ socio-demographic background included ethnicity, age, marital status, education, and years working as a MDW in Hong Kong.

### Outcome measures

Anxiety was measured using the Generalized Anxiety Disorder 7-item scale (GAD-7) [34]. Respondents were asked the frequency of negative feelings such as trouble relaxing, and feeling easily annoyed or irritable in the past two weeks on a 4-point scale. The score ranges from 0 to 21 with a higher score indicating higher anxiety. The standardized Cronbach’s alpha in the English and Indonesian versions were 0.89 and 0.86 for GAD-7, respectively.

Depressive symptoms were measured using the Patient Health Questionnaire 9-item scale (PHQ-9) [35]. Respondents were asked the frequency of negative feelings such as feeling down, depressed, and hopeless in the past two weeks on a 4-point scale. The score ranges from 0 to 27 with a higher score indicating more depressive symptoms. The standardized Cronbach’s alpha in the English and Indonesian versions were 0.83 and 0.84 for PHQ-9, respectively.

Both outcome measures have been shown to be reliable, and were previously validated among Filipino and Indonesian MDWs in Macao SAR, China [36, 37]. Different cut-off points have been recommended to assess probable anxiety and depression. Generally, a score of ≥ 10 has been recommended for both GAD-7 [34] and PHQ-9 [35]. However, a cut-off of ≥ 7 for GAD-7 and ≥ 6 for PHQ-9 has also been recommended specifically among the female MDW population [37].

### Statistical analysis

In case of double entries by the same participants in both the first and second efforts of data collection, we removed the second set of data. Double entries were detected by searching for duplicate phone numbers, at which point the participant was contacted to enquire on reason for double entry. For the data analysis, cases were first weighted to reflect the population of female MDWs by age group according to the 2020 Hong Kong census [26]. Next, descriptive statistics were computed to display the frequency of each variable as well as the mean and standard deviation (SD) of anxiety and depression scores. Unadjusted OLS linear regression was performed between each variable and each outcome measure to assess their association. Background variables which had a *p*-value ≤ 0.2 were included as covariates in the mediation analysis.

A parallel mediation analysis proposes that two or more variables independently mediate the relationship between an independent and dependent variable. This parallel mediation analysis included employment conditions score as independent variable, anxiety or depression as outcome, and stress and job satisfaction as mediators. Hayes’s method for a multicategorical X variable was used [38]. Bootstrapping with 5000 resamples was used to test the significance of the indirect effects where 95% confidence intervals that do not cross 0 are considered significant. Eligible covariates were included in the model to control for potential confounders. All analyses were completed with SPSS Version 26, while mediation analysis was completed using the PROCESS macro for SPSS [39].

## Results

### Descriptive statistics

The frequencies and percentages of background characteristics, mediating variables, and probable anxiety and depression are presented in Table 2. After removing 33 double entries and one survey with no response to the outcome variables, a total of 1,965 responses were considered, of which, 1,584 (80.6%) were Filipino and 381 (19.4%) were Indonesian nationals. Almost half of respondents were aged 35–44, while 40.6% reported attending “up to secondary school”, and nearly one-quarter reported working in Hong Kong for 10 or more years. Experience of some level of stress was reported by 63.0% of respondents, while 12.7% reported having a low job satisfaction. 17.7% had probable anxiety, while 30.8% had probable depression. When assessing probable anxiety and depression with a higher cut-off of ≥ 10 [34, 35], the rates decreased to 6.0% and 8.6%, respectively (data not shown separately).

**Table 2:** Characteristics of the study sample (*n* = 1965)

Variables	Weighted %	Unweighted n	Anxiety Mean (SD)	Depression Mean (SD)
**Total**		1965	3.1 (3.8)	3.9 (4.0)
**Background characteristics**				
Ethnicity				
Filipino	80.6%	1601	3.2 (3.8)	4.0 (4.0)
Indonesian	19.4%	364	2.5 (3.6)	3.5 (4.1)
Age				
20–34	35.4%	691	3.7 (4.0)	4.6 (4.1)
35–44	44.7%	954	2.9 (3.6)	3.8 (3.9)
40+	19.8%	320	2.3 (3.6)	3.2 (3.8)
Marital status				
Never married/divorced/widowed/separated	53.0%	1045	3.1 (3.8)	3.9 (4.0)
Married	47.0%	920	3.1 (3.8)	3.9 (4.0)
Education level (highest level attended)				
Secondary school	40.6%	799	2.8 (3.7)	3.6 (4.0)
Technical or vocational school	27.5%	535	3.3 (3.7)	4.1 (3.9)
University or postgraduate degree	31.9%	631	3.3 (3.8)	4.3 (4.0)
Years working as MDW in Hong Kong				
1–3	34.2%	661	3.6 (3.9)	4.2 (4.0)
4–9	42.2%	882	3.2 (3.7)	4.2 (4.0)
10+	23.6%	422	2.3 (3.5)	3.1 (3.7)
**Psychosocial factors**				
How often do you feel stress these days?				
Not at all	37.0%	717	1.1 (2.1)	1.5 (2.4)
Only a little	38.8%	764	3.3 (3.2)	4.5 (3.4)
To some extent	12.6%	255	5.1 (3.9)	6.0 (3.5)
Rather much	4.3%	82	6.3 (4.4)	7.4 (4.4)
Very much	7.3%	147	6.8 (5.6)	7.9 (5.8)
Generic Job Satisfaction Scale^a^				
Low job satisfaction	12.7%	241	4.7 (4.1)	5.7 (4.6)
Average job satisfaction	24.3%	497	4.1 (4.2)	5.0 (4.3)
High job satisfaction	62.9%	1227	2.4 (3.3)	3.2 (3.5)
**Mental health**				
Probable anxiety (Cut-off ≥ 7)*	17.7%	355	9.5 (3.5)	9.0 (4.3)
Probable depression (Cut-off ≥ 6)*	30.8%	619	6.6 (4.2)	8.8 (3.2)
Comorbidity	15.1%	301	9.7 (3.7)	10.0 (4.0)

^a^ Items in Generic Job Satisfaction scale: (1) “I receive recognition for a job well done,” (2) “I feel close to the people at work,” (3) “I feel good about working at this household,” (4) “I feel secure about my job,” (5) “I believe the family is concerned about me,” (6) “on the whole, I believe work is good for my physical health,” (7) “my wages are good,” (8) “all my talents and skills are used at work,” (9) “I get along with my supervisors,” (10) “I feel good about my job”. Score range from 10 to 50 where 39–50 is high satisfaction, 32–38 is average satisfaction, and ≤ 31 is low satisfaction. *As recommended by Garabiles et al. 2020

### Employment conditions score characteristics

The frequencies of each item in the ECS and mean and SD scores for anxiety and depression are shown in Table 2. Over a quarter (27.2%) of respondents reported experiencing four or more of the 17 conditions. The five most commonly reported ECS items were ever needing to work before being able to begin day off (31.9%), having overcrowded home environments or lack of privacy (26.9%), not always getting enough to eat (24.1%), ever being woken up and asked to work in the middle of the night (23.2%), and working on average 16 or more hours per day in the past month (20.8%).

### Unadjusted OLS linear regression

All variables, except marital status, met the required level of significance (*p* < 0.2) to be included in the mediation analysis for both the anxiety and depression outcomes, as seen in Table 3. When compared with good ECS, both average ECS (anxiety: β_unstandardized_ = 1.21, 95% CI 0.83, 1.60, *p* < 0.001; depression: β_unstandardized_ = 1.56 95% CI 1.15, 1.96, *p* < 0.001) and poor ECS (anxiety: β_unstandardized_ = 3.11, 95% CI 2.68, 3.54, *p* < 0.001; depression: β_unstandardized_ = 3.56, 95% CI 3.10, 4.01, *p* < 0.001) were significant associated with increases in anxiety and depression scores.

**Table 3:** OLS linear regression estimates of Anxiety and Depression scores (*n* = 1965)

	Anxiety		Depression	
	β (95% CI)	*p*-value	β (95% CI)	*p*-value
**Background characteristics**				
Ethnicity				
Filipino	Ref.		Ref.	
Indonesian	-0.75 (-1.17, -0.33)	< 0.001	-0.50 (-0.95, -0.06)	0.028
Age		< 0.001		< 0.001
20–34	Ref.		Ref.	
35–44	-0.75 (-1.11, -0.39)		-0.79 (-1.17, -0.41)	
45+	-1.33 (-1.80, -0.86)		-1.39 (-1.89, -0.89)	
Marital status				
Never married/divorced/widowed/separated	Ref.		Ref.	
Married	-0.07 (-0.41, 0.26)	0.676	0.01 (-0.34, 0.36)	0.955
Education level		0.005		0.002
Up to secondary school	Ref.		Ref.	
Technical or vocational school	0.59 (0.18, 0.99)		0.56 (0.13, 0.98)	
University or postgraduate degree	0.52 (0.13, 0.90)		0.67 (0.26, 1.07)	
Years working as MDW in Hong Kong		< 0.001		< 0.001
1–3	Ref.		Ref.	
4–9	-0.39 (-0.76, -0.02)		-0.08 (-0.47, 0.31)	
10+	-1.24 (-1.68, -0.79)		-1.07 (-1.53, -0.60)	
**Psychosocial factors**				
How often do you feel stress these days?	1.59 (1.47, 1.72)	< 0.001	1.75 (1.62, 1.88)	< 0.001
Generic Job Satisfaction Scale	-0.12 (-0.15, -0.10)	< 0.001	-0.13 (-0.16, -0.10)	< 0.001
**Employment conditions score**		< 0.001		< 0.001
Good (0)	Ref.		Ref.	
Average (1–3)	1.21 (0.83, 1.60)		1.56 (1.15, 1.96)	
Poor (≥ 4)	3.11 (2.68, 3.54)		3.56 (3.10, 4.01)	

Universal *p*-values given for multicategorical variables. Both stress (1–5) and job satisfaction (10–50) were assessed as continuous variables. All β are displayed as unstandardized coefficients

### Parallel mediation models

**Fig. 2:**
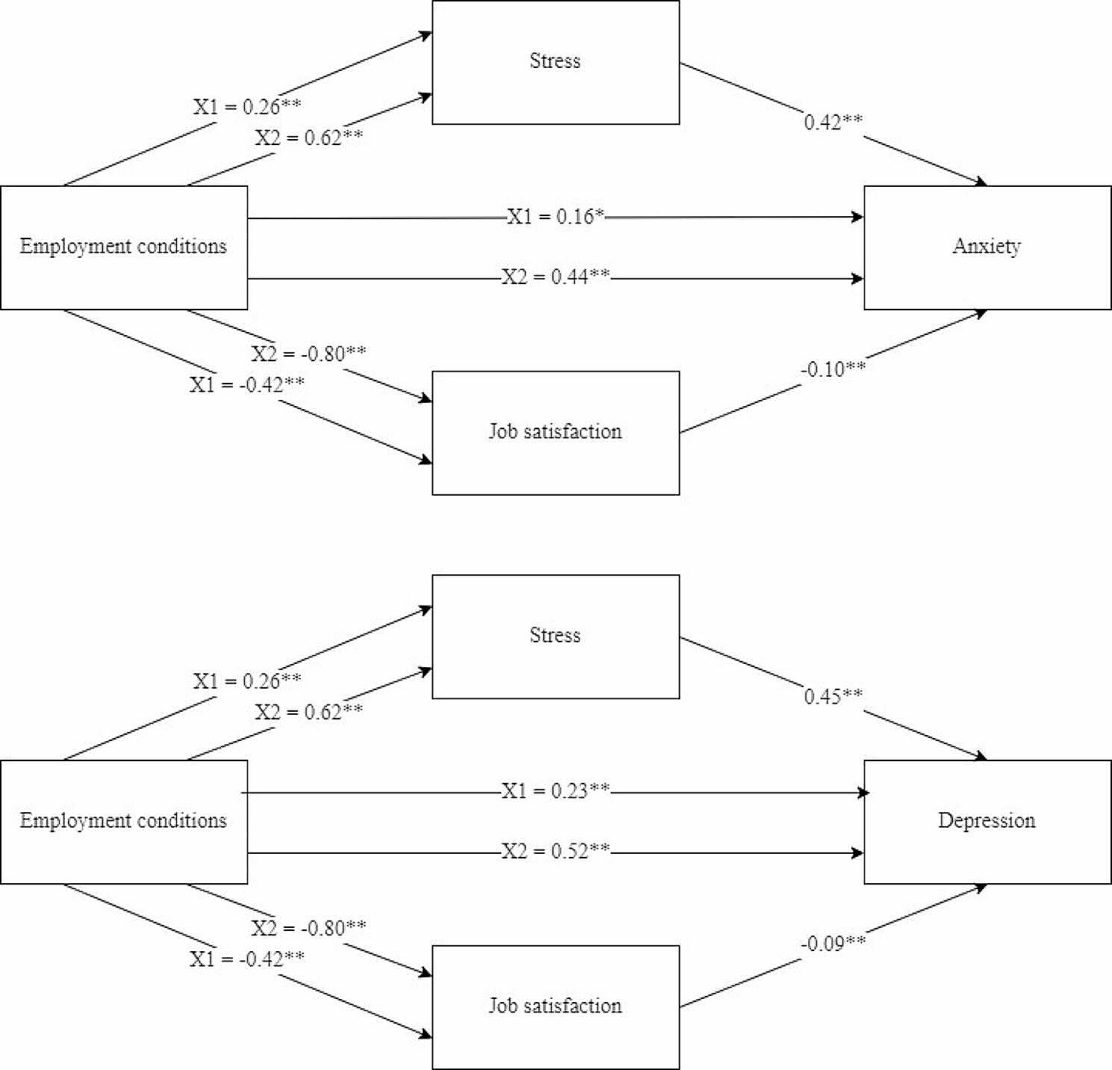
Parallel mediation analysis *p < 0.01; **p < 0.001. X1 ECS = Good ECS vs. Average ECS; X2 ECS = Good ECS vs. Poor ECS. Potential confounders: ethnicity, age, marital status, education level, years working in Hong Kong. All coefficients are standardized

The parallel mediation analysis found average and poor ECS, when compared with good ECS, to be indirectly associated with both anxiety and depressive symptoms through their relationship with stress and job satisfaction, when adjusted for background characteristics. The indirect effects, 95% bootstrapped CIs, total effects, and direct effects for both models are shown in Table 4, while the coefficients for each pathway in both models are illustrated in Fig. 2.

**Table 4:** Parallel mediation of employment conditions with stress and job satisfaction on anxiety and depression (*n* = 1965)

Total Effect	Direct Effect	Relationship	Indirect effect	Boot LLCI	Boot ULCI
X1 ECS → Anxiety	X1 ECS → Anxiety	X1 ECS → Stress → Anxiety	0.108	0.068	0.151
0.304 (< 0.001)	0.156 (0.001)	X2 ECS → Stress → Anxiety	0.261	0.207	0.319
X2 ECS → Anxiety	X2 ECS → Anxiety	X1 ECS → Job satisfaction → Anxiety	0.040	0.022	0.064
0.782 (< 0.001)	0.444 (< 0.001)	X2 ECS → Job satisfaction → Anxiety	0.077	0.043	0.116
X1 ECS → Depression	X1 ECS → Depression	X1 ECS → Stress → Depression	0.114	0.071	0.158
0.376 (< 0.001)	0.225 (< 0.001)	X2 ECS → Stress → Depression	0.275	0.217	0.335
X2 ECS → Depression	X2 ECS → Depression	X1 ECS → Job satisfaction → Depression	0.037	0.018	0.062
0.866 (< 0.001)	0.520 (< 0.001)	X2 ECS → Job satisfaction → Depression	0.071	0.035	0.113

ECS = employment conditions score; X1 ECS = Good ECS vs. Average ECS; X2 ECS = Good ECS vs. Poor ECS; LLCI = lower limit confidence interval; ULCI = upper limit confidence interval. Potential confounders: ethnicity, age, education level, years working in Hong Kong. All coefficients are standardized

When compared with good ECS, those with average ECS and poor ECS had increased levels of stress, and an increase in stress was associated with an increase in anxiety and depressive symptoms (Fig. 2). Additionally, when compared with good ECS, those with average and poor ECS had a decrease in job satisfaction, while an increase in job satisfaction significantly decreased anxiety and depressive symptoms. The 95% bias-corrected CIs based on bootstrap analysis (Table 4) demonstrate the indirect effect through which both stress and job satisfaction with confidence intervals above zero. Both direct effects of ECS on anxiety and depressive symptoms were also statistically significant.

The linear model of each pathway’s residuals was approximately normally distributed and the scatter plot of residuals versus predicted values showed no evidence of heteroscedasticity. Further, the variance inflation factors for the model showed no evidence of multicollinearity (VIF < 5).

## Discussion

To the best of our knowledge, the present study is the first of its kind to demonstrate evidence of the cumulative effect of negative employment conditions on anxiety and depressive symptoms in a large sample of female MDWs. While controlling for sociodemographic factors, we found an increase in negative employment conditions to be directly associated with an increase in anxiety and depressive symptoms. Furthermore, there was a significant indirect effect on these associations through stress and job satisfaction. The findings add new knowledge in the literature by displaying the cumulative effect that negative employment conditions may have on the mental health among live-in MDWs.

### State of employment conditions

There is little change in the reported employment conditions experiences amongst MDWs, which are consistent with previous studies in Hong Kong from 2015 to 2017 [14, 40]. Our sample had a longer average time since migration of 6.8 years, which may be a contributing factor to some employment differences [14, 40]. Our sample reported an average of 12.6 working hours a day (i.e., 75.6 h/week for six working days, not shown separately), which is consistent with previous studies [40]. Additionally, over 20% reported working 16 or more hours per day, highlighting the employer exploitations especially when there is no limit on weekly working hours. Hong Kong generally has one of the longest average working hours per week globally, at 42 h in 2018; however, our MDWs approximately worked an additional 33 h weekly on average [41]. While only 2.1% of our sample reported not receiving a food allowance nor food provision, as required by law [42], about 24% reported not having enough food to eat for their daily meals. This discrepancy indicates that those being provided food by their employers, while legal, may be inadequate, and warrants further investigation. Furthermore, local law stipulates that MDWs should be granted all statutory holidays, one 24-hour period off per week, and clear agreement of job duties as written in the contract [42]. However, 4.9%, 15.3%, and 6.7% of our study sample reported they were not provided with statutory holiday benefits, 24-hour off per week and formal agreement of work responsibilities. In other words, despite legal protections, MDWs remain vulnerable and deprived of their basic rights. The physical living standard was also concerning with respondents reporting overcrowding and lack of privacy, dirty living conditions, and a lack of electricity or plumbing. MDWs enduring these conditions often have no choices but remain resilient in order to provide financial security for themselves and their family [43].

### Mediating factors

While both stress and job satisfaction mediated the effect of employment conditions on anxiety and depressive symptoms, the mediating effect through stress was stronger; suggesting strategies to address stress may prove more effective in reducing mental health severity. For instance, in addition to the work environment as a primary stressor, additional risk factors included not being part of a social network in the host country and living away from family [13, 25, 44]. Studies assessing stress management and coping strategies among female MDWs reported setting aside personal time for oneself, control of thoughts and emotions, and religion to be the primary coping mechanisms or strategies [25]. However, policies must be implemented to provide the ability for MDWs to implement these strategies, especially the need for adequate time and privacy [25].

The indirect effect that job satisfaction had on anxiety and depressive symptoms may be because MDWs know the reality of their difficult job situation and have less expectation of high job satisfaction. A study of MDW caregivers in Hong Kong found high job satisfaction among those who had been in Hong Kong longer, could speak the local language (Cantonese), and had more satisfaction in their living conditions [24]. This study suggests that those better integrated into the local culture had better job satisfaction, and potentially, better mental health outcomes. Better local cultural integration may also lessen communication barriers between the employee and employer, creating a less stressful work environment. While many MDWs learn basic Cantonese language skills prior to arriving in Hong Kong, it may be beneficial for them be provided with additional courses in language and culture after arriving in Hong Kong.

### Employment conditions and mental health

The significant increase in anxiety and depressive symptoms for those experiencing average and poor ECS compared with good ECS points to the negative cumulative effect negative employment conditions have on MDWs. The significant direct effects from those with average and poor ECS on both mental health outcomes provides evidence to support the need for addressing these conditions in policymaking. Weekly working hours and an adjustment to the live-in requirement need to be considered to ensure basic protection of their health and well-being.

As mentioned above, the average MDW in Hong Kong works significantly longer than the average local worker, and for much less pay. These contrasting circumstances have been rationalized because of the domestic “nature” of MDWs employment and the free room and board they should be provided as part of their contract. Nevertheless, our findings reported 4.8% and 24.0% of respondents slept in places such as the kitchen and not always having enough food to eat, respectively. There is precedent for a weekly working hours cap as the neighboring Macao allows for a weekly working hours agreement in their MDWs’ employment contract [45]. An adjustment to the live-in requirement, although logistically challenging, warrants serious consideration given its potential positive impact. Given Hong Kong’s geographical constraints in terms of land availability, building new housing is limited and expensive which propels rental fees high [46]. To overcome these barriers, introduction of a live-out option could be made possible if boarding houses were built specifically for MDWs at a lower rental price. A pilot program could house MDWs in a boarding house to assess whether this improves their living conditions and well-being. This could also give employers who value privacy an option to the mandatory live-in rule. Additionally, a mandatory household assessment of a potential employer prior to authorization to hire a MDW could be implemented to ensure essential living conditions.

As policy changes take considerable time, in the short term, increased availability to mental health services among MDWs could be a first step in making improvements. Current mandatory health insurance provided by the employer does not cover mental health services [47]; so, MDWs have traditionally relied on family and friends, non-governmental organizations, and church communities for mental health support [25]. However, some studies suggest support from peers may have an adverse effect as peers are also experiencing similar stressors [13, 48]. Professional mental health services may be a more effective approach. Providing of electronic mental health services, as a study in Macao found to be a promising tool [49], may be replicated in Hong Kong. However, MDWs were found less likely to take up COVID-19 vaccinations [50] and free HIV/syphilis testing [51] when they had long working hours, which could deter them from seeking needed healthcare. Therefore, allocation of personal time and space is necessary to enable access and use of such services.

### Limitations and future research

It is important to acknowledge the data collection and sampling biases when considering the findings of this study. First, the additional non-probability sampling approach and selection biases did not allow us to provide population prevalence estimates. Nearly two-thirds of the study sample were drawn based on a non-probability approach - they were somewhat younger and less educated than the randomly sampled respondents. However, we computed sample weights by age to better represent the population of female MDWs in Hong Kong. Second, the data collection period, spanning one year, took longer than anticipated due to the COVID-19 pandemic. However, Hong Kong maintained consistent social distancing restrictions over this period with relatively low daily case counts of COVID-19, minimizing potential confounding factors from the pandemic. Third, as this study used a cross-sectional design, we cannot establish casual inference. Fourth, we collected substantially more surveys from Filipinos than Indonesians, which may bias rates of probable anxiety and depression in the reports of Indonesian community. However, this would not influence the association found in the analysis. Fifth, only an English version was provided to Filipino MDWs after consideration from the pilot survey. While this was done due to Filipino MDWs expressing preference for the English version, this may have prevented some Filipino MDWs from completing our survey. Finally, as there is not an internationally recognized MDW employment conditions instrument available, our study’s findings are not directly comparable with previous literature. Future studies should consider a longitudinal and life course approach, preferably from pre-migration to post-migration, to establish causal inference between employment conditions and health.

## Conclusions

Among a large sample of live-in female MDWs in Hong Kong, we found that an increase in negative employment conditions significantly increased anxiety and depressive symptoms, while stress and job satisfaction mediated this effect. Our findings have implications for policymakers to consider possible changes in employment regulation for MDWs including a weekly working hours’ limit and increased scrutiny of employment conditions to better safeguard the health and well-being of MDWs.

### Electronic supplementary material

Below is the link to the electronic supplementary material.

**Supplementary Table 1**. Spearman correlation coefficients between the variables in the employment conditions score (n = 1965)

## Data Availability

The data that support the findings of this study are available on request from the corresponding author, RYC.

## Abbreviations

MDW: Migrant domestic worker
ECS: Employment conditions score
IQR: Interquartile range
GJSS: Generic job satisfaction scale
GAD-7: Generalized anxiety disorder – 7
PHQ-9: Patient health questionnaire – 9
SD: Standard deviation
VIF: Variance inflation factor
