# Supplementary material for: Employment conditions and mental health of overseas female migrant domestic workers in Hong Kong: a parallel mediation analysis
Source: Int J Equity Health. 2024 Jan 17;23:8. doi: 10.1186/s12939-024-02098-3 (PMC10792881; doi:10.1186/s12939-024-02098-3)
Supplement: Supplementary file 1 — Supplementary Table 1. Spearman correlation coefficients between the variables in the employment conditions score (n = 1965) [file 12939_2024_2098_MOESM1_ESM.docx]

**Supplementary Table 1.** Spearman correlation coefficients between the variables in the employment conditions score (n = 1965)

|  | 1 | 2 | 3 | 4 | 5 | 6 | 7 | 8 | 9 | 10 | 11 | 12 | 13 | 14 | 15 | 16 | 17 |
| --- | --- | --- | --- | --- | --- | --- | --- | --- | --- | --- | --- | --- | --- | --- | --- | --- | --- |
| (1) Sleeping arrangement | 1 |  |  |  |  |  |  |  |  |  |  |  |  |  |  |  |  |
| (2) At your employer’s home, is there overcrowding or lack of privacy? | 0.194* | 1 |  |  |  |  |  |  |  |  |  |  |  |  |  |  |  |
| (3) At your employer’s home, is it unsanitary, pest infested, or poorly ventilated? | 0.147* | 0.326* | 1 |  |  |  |  |  |  |  |  |  |  |  |  |  |  |
| (4) At your employer’s home, is there any lack of water, electricity, or plumbing? | 0.082* | 0.282* | 0.285* | 1 |  |  |  |  |  |  |  |  |  |  |  |  |  |
| (5) Do you receive a food allowance or have food provided? | 0.064* | 0.105* | 0.126* | 0.100* | 1 |  |  |  |  |  |  |  |  |  |  |  |  |
| (6) Did you always get enough food to eat? | 0.099* | 0.154* | 0.191* | 0.115* | 0.189* | 1 |  |  |  |  |  |  |  |  |  |  |  |
| (7) Did you ever have your salary withheld or given less? | 0.027 | 0.140* | 0.188* | 0.081* | 0.082* | 0.127* | 1 |  |  |  |  |  |  |  |  |  |  |
| (8) Did you ever have your phone or internet taken away? | 0.065* | 0.147* | 0.163* | 0.115* | 0.048* | 0.146* | 0.218* | 1 |  |  |  |  |  |  |  |  |  |
| (9) Did your employer ever verbally abuse you? | 0.085* | 0.190* | 0.177* | 0.119* | 0.167* | 0.214* | 0.129* | 0.164* | 1 |  |  |  |  |  |  |  |  |
| (10) Did your employer ever physically or sexually abuse you? | 0.044 | 0.083* | 0.128* | 0.054* | 0.116* | 0.091* | 0.105* | 0.122* | 0.149* | 1 |  |  |  |  |  |  |  |
| (11) Did your employer ever take away your day off? | 0.091* | 0.206* | 0.229* | 0.119* | 0.133* | 0.206* | 0.266* | 0.252* | 0/273* | 0.119* | 1 |  |  |  |  |  |  |
| (12) Did your employer give you all statutory holidays? | 0.005 | 0.086* | 0.055* | 0.058* | 0.032 | 0.033 | 0.058* | 0.047* | 0.134* | 0.055* | 0.159* | 1 |  |  |  |  |  |
| (13) Did your employer ever restrict you from going outside? | 0.051* | 0.087* | 0.089* | 0.040 | 0.036 | 0.065* | 0.059* | 0.108* | 0.177* | 0.003 | 0.130* | 0.154* | 1 |  |  |  |  |
| (14) Did your make you do work beyond what is stated in the contract? | 0.083* | 0.206* | 0.179* | 0.113* | 0.072* | 0.131* | 0.133* | 0.171* | 0.190* | 0.129* | 0.226* | 0.110* | 0.167* | 1 |  |  |  |
| (15) Past month average daily working hours | 0.033 | 0.109* | 0.114* | 0.068* | 0.060* | 0.181* | 0.071* | 0.073* | 0.120* | 0.031 | 0.106* | 0.047* | 0.036 | 0.095* | 1 |  |  |
| (16) Were you ever woken in the middle of the night and asked to work? | 0.063* | 0.194* | 0.156* | 0.090* | 0.069* | 0.230* | 0.080* | 0.159* | 0.250* | 0.110* | 0.252* | 0.114* | 0.138* | 0.216* | 0.220* | 1 |  |
| (17) Did you ever have to work before being allowed to begin your day off? | 0.092* | 0.210* | 0.184* | 0.091* | 0.057* | 0.216* | 0.133* | 0.146* | 0.277* | 0.062* | 0.342 | 0.098* | 0.146* | 0.193* | 0.159* | 0.297* | 1 |

*p < 0.05
